# Seroprevalence and Public Health Significance of Toxoplasmosis in Small Ruminants of Pastoral Community in Yabello District, Borana Zone, Southern Ethiopia

**DOI:** 10.1155/2021/6683797

**Published:** 2021-05-18

**Authors:** Kula Jilo, Dechassa Tegegne, Sadik Kasim, Golo Dabasa, Wubishet Zewdei

**Affiliations:** ^1^Veterinary Epidemiology, Addis Ababa University, College of Agriculture and Veterinary Medicine, P.O. Box. 34, Bishoftu, Ethiopia; ^2^Veterinary Biotechnology, Jimma University, College of Agriculture and Veterinary Medicine, Jimma, Ethiopia; ^3^Yabello Regional Veterinary Laboratory, P.O. Box. 569, Yabello, Ethiopia

## Abstract

Toxoplasmosis is a zoonotic protozoan disease. Data on seroepidemiology of toxoplasmosis in Ethiopia is scarce, almost null in the pastoral area of the Borana zone. The study was carried out to determine the seroprevalence, to identify risk factors of toxoplasmosis in sheep and goats, and to assess the awareness level of pastoralists about toxoplasmosis in the Yabello district of Borana zone, Southern Ethiopia. A cross-sectional study was conducted from November 2016 to April 2017 in six peasant associations of the Yabello district of Borana zone, Southern Ethiopia. A total of 400 serum samples of randomly selected small ruminants owned by pastoralists were examined to detect antibodies specific to *Toxoplasma gondii* using Latex Agglutination Test (SPINREACT, Girona, Spain). A semistructured questionnaire survey was used to conduct a face-to-face interview with owners (*n* = 100) of sampled flocks. Logistic regression analysis was used to determine the association of hypothesized risk factors. The overall seroprevalence was 52.8% of which 57.8 and 47.8% were sheep and goats, respectively. Univariate logistic regression analysis revealed a higher seroprevalence ratio of *T. gondii* infection in sheep than goats (COR: 1.95, 95% CI: 1.226–3.112; *P* = 0.005). Multivariate logistic regression analysis indicated significantly higher odds of acquiring *T. gondii* infection in adult animals (sheep: (AOR = 2.26, 95% CI: 1.323–3.874; *P* = 0.003), goats: (AOR = 2.15; 95% CI: 1.009–4.579; *P* = 0.047)), female sheep (AOR = 2.45; CI: 1.313–4.568; *P* = 0.005), animals from lowland areas (sheep: (AOR = 2.28; CI: 1.190–4.356; *P* = 0.013), goat: (AOR = 3.27; CI: 1.386–7.723; *P* = 0.007)), animal drinking lake water (sheep: (AOR = 1.93; CI: 1.011–3.698; *P* = 0.046), goat: (AOR = 2.96; CI: 1.297–6.771; *P* = 0.010)), and goats with history of abortion (AOR = 2.42; CI: 1.242–4.711; *P* = 0.009) than young animals, male (sheep), animals from midland areas, animals drinking wells water, and flock with no history of abortion (goat), respectively. Among respondents, 97.0% had no knowledge about toxoplasmosis and 75.0% drink raw milk and consume the meat of sheep and goats. 80.0% of respondents had no knowledge about the risk of cats to human and animal health while 70.0% of them had domestic cats and practice improper fetal body handling. Highly prevailing toxoplasmosis in small ruminants of the Yabello district might pose a serious economic loss and be a potential public health threat to the extremely vulnerable pastoralists. Therefore, awareness and further studies are warranted to tackle the economic and public health consequences of *T. gondii* infection.

## 1. Introduction

Protozoan parasites are major challenges in the development of the livestock industry. Among these, toxoplasmosis is a worldwide zoonotic disease caused by an obligate intracellular parasite, *Toxoplasma gondii* [1]. The major morphological forms of *T. gondii* are oocyst, trophozoite, and tissue cyst which favor warm climate and humid areas for sporulation and survival [2]. *T. gondii* has a broad spectrum host range, affecting all vertebrate animals including human beings [3]. Cats and wild felids are the only definitive hosts that shed oocysts with their feces to the environment while humans and other animals act as intermediate hosts [4]. Cats acquire infection by consumption of cysts present in the tissues of the infected intermediate hosts or via ingestion of oocysts with food or water [5]. However, intermediate hosts harbor *T. gondii* through consumption of viable cysts in undercooked meat, unpasteurized milk, and food and water contaminated with oocysts and congenitally during pregnancy [6–8].

Toxoplasmosis is a disease of economic importance in the livestock industry causing reproductive problems such as stillbirths, abortions, postnatal mortality, and fetal malformations in farm animals [6, 9]. *T. gondii* pose a serious risk of public health for zoonotic transmissions [5]. In humans, toxoplasmosis is a leading cause of death from foodborne illness in a group of five parasitic diseases that have been targeted by the Center for Disease Control and Prevention (CDC) for public health measures [10]. It is highly prevalent in farm animals and nearly one-third of the human population is chronically infected with *T. gondii* [6, 7, 9]. It causes a major problem in immunocompromised individuals and pregnant women causing abortion, encephalitis, schizophrenia, epilepsy, lymphadenopathy, and ocular [5, 11–13]. Toxoplasmosis is commonly diagnosed serologically by the detection of antitoxoplasma antibodies in serum or by the isolation of the agent from tissues of infected individuals [1, 14]. Currently, there is no satisfactory treatment and effective vaccine against *T. gondii*, but avoiding consumption of raw meat, providing cooked meat for cats, and properly disposing cat feces are major prevention and control mechanisms [15, 16].

In Ethiopia, different reports revealed a widespread occurrence of toxoplasmosis in farm animals and humans. Seroprevalence of 82.0% in goats from South Omo using microscopic agglutination test (MAT) [17], 58% in sheep from Jimma using Latex Agglutination Test (LAT) [18], and 96.7% in humans using ELISA [19] was reported. As compared to other African countries, the highest prevalence of *T. gondii* in farm animals was reported in Ethiopia [20]. However, even though a few studies attempted to know seroprevalence and associated risk factors of toxoplasmosis in Ethiopia, toxoplasmosis is an emerging foodborne parasitic disease and one of the most globally widespread zoonoses with considerable health and economic impacts [21]. Even though a few studies have been attempted to know seroprevalence and associated risk factors of toxoplasmosis in Ethiopia, they were largely restricted to central highlands for accessibility, availability of infrastructures, and the assumption behind the biology of *T. gondii* and the longer viability of *T. gondii* oocyst in moist and humid environments.

Besides the current seroprevalence survey of *T. gondii* in camel by Gebremedhin et al. (2016), there was no attempt in the Borana zone toward toxoplasmosis which is being reported from different parts of Ethiopia as one of the diseases causing huge economic loss and public health problem. Consumption of raw meat and milk is popular in Ethiopia particularly in pastoral areas of the Borana community. In addition, cats and dogs are reared as an integral part of household and livestock for the protection of rodents and predators' pastoral community. With their saying of “no dog, no sheep or goat,” dogs are used as second flock men and inseparable from the flock throughout their life. Moreover, the intimate relationship between humans and animals and the usage of an unimproved water source may facilitate the acquisition of *T. gondii* in pastoral areas. The worst, in pastoral areas, routine husbandry activities like sick animal care, milking, and cleaning barns are exclusively undertaken by women that may exacerbate the risk of *T. gondii* infection in pregnant women of the pastoral community. Although *T. gondii* oocysts survive more in warm and humid environments, there was no single study conducted on seroprevalence and associated risk factors of toxoplasmosis in small ruminates in the Borana zone. Therefore, the objectives of this study were to determine seroprevalence, to assess associated risk factors of *T. gondii* in small ruminants, and to assess the awareness level of pastoralists in the Yabello district.

## 2. Methods

### 2.1. Study Area

The study was conducted from November 2016 to April 2017 in the Yabello district of Borana zone, Southern Ethiopia. Yabello town, the capital of the Borana zone, is located 570 km south of Addis Ababa. It has a latitude and longitude of 4°53′N 38°5′E and an elevation ranging from 350 to 1857 meter above sea level (masl). The annual mean daily temperature of the district ranges from 19 to 24°c. Agroecologically, the Yabello district is arid and semiarid having lowland and midland areas. The average annual rainfall ranges from 300 to 700 mm of which 65% is in April–June (locally called “*ganna*” and the remaining 35% is in September–November (locally called “*hagayya*”) with considerable spatial and temporal variability in quantities and distribution. An extensive pastoral livestock production system with mobility is the vital source of food and income for the livelihood of people while opportunistic cultivation is practiced around towns and in bottom valley areas where the soil moisture content stays high for a longer time. The dominant vegetation of this area is the savanna type. The total population of Yabello district is 125,233, and the livestock population composes 83,717 cattle, 42,491 sheep, 84,159 goats, and 18,613 camels [22].

### 2.2. Study Population

Population of interest was sheep and goats, which were owned by pastoralists who manage these animals for dual purpose (milk and meat) under extensive production system in communal grazing land in Yabello district. Additionally, pastoralists of Yabello district were also included for awareness study.

### 2.3. Study Design and Sample Size

A cross-sectional study design was applied to determine the seroprevalence of toxoplasmosis, to identify possible risk factors, and to assess the awareness level of pastoralists about toxoplasmosis in purposively selected Yabello district of Borana zone, Southern Ethiopia. Yabello district was selected for the availability of infrastructure and varied agroecology (lowland and midland). From a total of 19 pastoral associations (PAs), 3 PAs were selected from each lowland and midland area based on road accessibility. Similarly, Haro Bake, Darito, and Cholkasa from lowland and Dida Yabello, Obdaya, and Areri PAs from midland were sampled. A total of 384 samples was initially obtained using a 95% level of confidence interval (CI) and 5% desired level of precision with the assumption of 50% expected prevalence of toxoplasmosis in small ruminants in the study area based on the formula given by [23]: *N*=1.962 × *P*_exp_(1 − *P*_exp_)/*D*^2^, where *N* is the required sample size, *P*_exp_ is the expected prevalence, and *D*^2^ is the desired absolute precision. However, for the sake of precision, 400 samples were collected.

### 2.4. Blood Collection

A simple random sampling technique was applied for both species to collect blood (approximately 5 ml) from study animals. Conveniently, 6 and 10 samples were collected from small (<50 animals) and large flocks (>50 animals). The jugular veins of animals were aseptically bled using 10 ml plain vacutainer tubes, vein puncture needle, and needle holder. Vacutainer tubes were properly labeled with waterproof marker with the necessary information. Blood samples were dispatched to Yabello regional veterinary laboratory for serological assay.

### 2.5. Serological Examination

Blood samples were allowed to stay 24 hours at room temperature to coagulate and then centrifuged at 3000 RPM for 10 minutes. Serum was decanted into a new and labeled vacutainer tube and stored at −20°C in the refrigerator until a qualitative serological test was performed. Serum was tested for the presence of anti-*T. gondii* antibodies by Toxo Latex Agglutination Slide Test (LAT). The test was done according to the manufacturer's instructions (SPINREACT, S.A/S.A.U Ctra Santa Coloma, Girona, Spain). LAT is a slide agglutination test for qualitative and semiquantitative detection and standardized to detect more than 10 IU/ml anti-*T. gondii* antibodies. The presence of a visible agglutination indicates the presence of anti-*T. gondii* antibodies in the samples tested. Latex particles coated with soluble *T. gondii* antigen are agglutinated when mixed with samples containing anti*-T. gondii* antibodies. The diagnostic sensitivity and specificity of the test are 96.1% and 89.6%, respectively. Positive Predictive Value (PPV) was calculated as the number of true positive results divided by all positive test results (true positive/true positive + false positive).

### 2.6. Questionnaire Survey

A semistructured questionnaire was prepared to gather information about the potential risk factors (both for animals and owners) and awareness of owners about toxoplasmosis. A questionnaire survey was randomly conducted face to face to interview 100 owners of flocks from which the blood sample was collected at the time of blood collection. During data collection, age, sex, water source, ecology, cat contact, history of abortion, and flock size of sampled animals were recorded. Additionally, residence place, educational status, consumption habit of milk and meat (cooked/raw), handling of animal wastes, hand wash practice, disposal system of animal wastes, a water source used for humans and animals, presence of domestic cats, and knowledge about toxoplasmosis were assessed.

### 2.7. Data Analysis

Data generated from the laboratory investigation and questionnaire survey was recorded in a Microsoft Excel spreadsheet (Microsoft Corporation) and coded and analyzed using SPSS version 20. The seroprevalence was calculated as the number of serologically positive samples divided by the total number of samples tested. Descriptive analysis was applied to describe the study population in relation to risk factors and variables. The association of the assumed risk factors and variables (species, sex, age, ecology, water source, cat contact, and flock size, and history abortion in the flock) for *T. gondii* seropositivity was analyzed by univariate logistic regression analysis. Variables with *P* value < 0.25 in univariable analysis were offered to the final multivariable model. Multicollinearity was checked using variance inflation factor (VIF), and the goodness of fit of the model was assessed by the Hosmer and Lemeshow test. The model was found fit for data and values of the Hosmer and Lemeshow test for final model for goat (*X*^2^ = 7.846; *P* = 0.346) and sheep (*X*^2^ = 7.025; *P* *=* 0.4260). By final multivariate logistic regression analysis, risk factors or variables having *P* < 0.05 were considered significant.

### 2.8. Ethical Considerations

The study was ethically approved by the ethical review board of Jimma University, College of Agriculture and Veterinary Medicine. The owners of the animals consent to use the animals in the study.

### 2.9. Quality Control and Precautions

For laboratory investigations, standard operating procedures and manufacturer's instructions were strictly followed. The quality of LAT was checked by both positive and negative controls.

## 3. Results

### 3.1. Overall Seroprevalence

In the present study, out of 400 small ruminants examined, 211 were found positive for anti-*T. gondii* antibodies with an overall seroprevalence of 52.8%. This study showed a seroprevalence of 57.8% (114/197) and 47.8% (97/203) in sheep and goats, respectively (Tables 1–3). The lowest seroprevalence and the highest seroprevalence were detected in Obdaya and Haro Bake (16.4 and 79.7%) PAs. Significant difference was observed in *T. gondii* seropositivity between two species of animals, and the odds of acquiring *T. gondii* was higher in sheep than goats (COR = 1.50; CI: 1.011–2.227; *P* = 0.044) (Table 3).

### 3.2. Sheep

By initial univariate logistic regression analysis, cat contact (*P* = 0.350) and flock size (*P* = 0.274) were dropped (scoring *P* *>* 0.25), whereas the rest variables which are sex (*P* = 0.001), age (*P* = 0.019), agroecology (*P* < 0.001), water source (*P* < 0.001), and the history of abortion (*P* = 0.100) were further analyzed with the multivariable model. By multivariate logistic regression analysis, the history of abortion was found not significant (*P* *=* 0.143) while sex (*P* = 0.005), age (*P* *=* 0.023), ecology (*P* = 0.013), and water source (*P* = 0.046) were found potential risk factors related to *T. gondii* infection in sheep. Thus, odds of acquiring *T. gondii* infection was significantly higher in female animals (AOR = 2.45; CI: 1.313–4.568; *P* = 0.005), adult animals (AOR = 2.13; CI: 1.137–5.618; *P* = 0.023), animals from lowland areas (AOR = 2.28; CI: 1.190–4.356; *P* = 0.013), and sheep that drank lake water (AOR = 1.93; CI: 1.011–3.698; *P* = 0.046) (Table 3).

### 3.3. Goats

In the univariate logistic regression analysis, sex (*P* = 0.069) was not significantly associated with *T. gondii* seropositivity (*P* > 0.25) (Table 4). In contrast, age (*P* = 0.047), agroecology (*P* = 0.007), water source (*P* = 0.010), history of abortion (*P* = 0.009), cat contact (*P* = 0.001), and flock size (*P* = 0.240) were positive predictors of seropositivity of *T. gondii* (*P* value <0.25) and further tested to determine multicollinearity effect with the multivariate logistic model. In multivariate logistic regression analysis, flock size (*P* = 0.132) and cat contact (*P* = 0.105) were dropped while age, agroecology, water source, and history of abortion in the flock were found potential predictors of *T. gondii* infection (*P* < 0.05) (Table 4). Thus, the odds of acquiring *T. gondii* infection was higher in adult animals (AOR = 2.15; CI: 1.009–4.579; *P* = 0.047), animals from lowland areas (AOR = 3.27; CI: 1.386–7.723; *P* = 0.007), sheep that drank lake water (AOR = 2.96; CI: 1.297–6.771; *P* = 0.010), and goats sampled from flocks with the history of abortion (AOR = 2.42; CI: 1.242–4.711; *P* = 0.009) (Table 4).

### 3.4. Questionnaire Survey

A questionnaire survey was randomly administered (face-to-face interview) to 100 flock herders/owners to assess the pastoral community's knowledge and perception about toxoplasmosis and potential risk factors. The result revealed that the pastoral community in the current study area is extremely at risk of toxoplasmosis and other zoonotic diseases due to combined effects of minimal awareness level, high illiteracy rate, insufficient improved water sources, overrated human-animal intimacy, poor hygienic conditions, and raw consumption habit of animal products.

The result showed that 90% of the respondents were rural residents and uneducated. From interviewees, 97% had no knowledge about toxoplasmosis. Regarding water sources for humans, only 5% of the respondents had access to pipe water. With respect to habit consumption, 93 and 80% of respondents consume raw milk and meat of small ruminants. Regarding fetus handling, only 33% of respondents perform handwashing after handling. Concerning domestic cat ownership and cat feeding practice, 70% had domestic cats and 67% fed their cat raw animal products whereas 80 and 53% had no knowledge about cats' risk to the animal and human health, respectively (Table 5).

## 4. Discussion

In Borana pastoral community, mutton and chevon are consumed usually as sheep and goats are slaughtered as a norm during cultural ceremonies, weddings, and women's birth giving as protein sources and for hosting of honorable guests. In this area, the milk of a small ruminant is consumed next to the milk of cattle. In particular, during severe droughts, when cattle productivity gets deteriorated, sheep and goats are a backup source of milk in pastoral areas since small ruminants can maintain productivity under an even harsh climate. Different studies have reported variable prevalence of *T. gondii* in animals from 0 to 100% in different parts of the world [24–27]. The difference in seroprevalence of *T. gondii* infection in small ruminants in different studies could be attributed to variation in environmental factors, cat density, management practice, species of the animals (species, age, sex, and breeds), serological tests used, the genetic background of the parasite and the host, the type of immune response elicited by the parasite, culture of the society, and different feeding habits which are some factors that contribute to the variation of seroprevalence of anti-*T. gondii* antibodies [6, 21, 28].

Previously, there was no report on seroprevalence of *T. gondii* infection in the Borana pastoral area except seroprevalence of *T. gondii* in camels (8.33%) [29]. However, the current study revealed a widespread occurrence of *T. gondii* infection in sheep (57.8%) and goats (47.8%) of Yabello district of Borana zone with significant difference between sheep and goats which might be due to the difference in feeding habit; browsing goats may have a lower chance of getting *T. gondii* infection than grazing sheep. In the current study, the seroprevalence of *T. gondii* antibody in sheep was 57.8% and comparable with the findings of [18, 30] who reported 56% and 58.73% from Central and Southwestern Ethiopia, respectively. However, a lower seroprevalence in sheep was reported from different parts of Ethiopia (31.6% and 31.59%), respectively [29, 31]. On the other hand, the current finding was higher than reports from other African countries such as Ghana (33.2%), Morocco (27.6%), Zimbabwe (47.5%), and Tunisia (10.85%) as reported by [32–35] whereas in this study lower seroprevalence of *T. gondii* antibody was recorded relative to others; 71%, 72.6%, 78%, 84.5%, and 95.7% were reported from Libya, Iran, Italy, Serbia, and Turkey, respectively [24, 36–39].

Seroprevalence of *T. gondii* antibodies in goats was 47.8% which was in agreement with the finding of 45.4% from central Ethiopia [30]; however, a lower prevalence of 15.48% and 19.30% was reported [40, 41]. In contrast, some scholars reported higher seroprevalence of *T. gondii* in goats (55.18%, 64%, and 79.5% respectively [17, 42, 43]). The seropositivity of the *T. gondii* antibody was significantly higher in female sheep than male sheep (AOR = 2.45; CI: 1.313–4.568; *P* = 0.005). This finding agreed with the findings of many scholars [17, 18, 30, 40]. The pastoral community keeps ewes for breeding to maximize the number of flocks which may increase the chance of *T. gondii* acquisition over time. Rams are usually sold for cash income to meet basic household requirements and frequently slaughtered for different purposes. Additionally, the hormonal difference in relation to the stress of lactation and pregnancy leading to immunosuppression may also increase susceptibility to toxoplasmosis in ewes [44]. In goats, there was no significant association between sex and seropositivity for *T. gondii* (*P* = 0.069) which is contrary to Tegegne [18] but matches other findings [40, 45]. This might be due to the feeding habit of goats and browsing, which may by default decrease the chance of infection and affect the distribution of toxoplasmosis between different sexes.

The likelihood of acquiring *T. gondii* infection was significantly higher in adult sheep than young sheep (AOR = 2.13; CI: 1.137–5.618; *P* = 0.023). This finding agreed with the previous reports [18, 40, 46]. Similarly, in goats, odds of acquiring *T. gondii* infection was higher in adults (AOR = 2.15; CI: 1.009–4.579; *P* = 0.047) which matched the results of many scholars [40, 45, 47]. Interestingly, the Borana community treats animals in different age groups in different manners. Before weaning, younger animals are kept and fed in separate houses made on pillars about 2 meters long above the ground where other animals or predators cannot reach. As a result, their chance of cat contact and contaminated soil or feed is very minimal. It is suggested that *T. gondii* infection of small ruminants is predominantly acquired postnatally after accessing food and water contaminated with oocysts [45, 47].

Altitude was significantly associated with seropositivity of *T. gondii* in the current Borana pastoral region. *T. gondii* oocysts survive more in a warm and humid environment. In the Yabello district, the annual mean temperature and humidity are 19–24°C and 48%, respectively. The occurrence of *T. gondii* infection was significantly higher in sheep from the lowland than that of midland areas (AOR = 2.28; CI: 1.190–4.356; *P* = 0.013). In the same manner, the odds of acquiring *T. gondii* infection were higher in goats from lowland areas than those from midland areas (AOR = 3.27, CI: 1.386–7.723; *P* = 0.007). This finding conceded with the finding from Mexico that reported a lower risk of toxoplasmosis at animals situated above 1200 meters above sea level (masl) argued that variation in cat density is a more potential determinant than altitude for the occurrence of *T. gondii* infection [48]. However, the current result was inconsistent with many previous studies that assured the relevance of higher altitude than lower altitude to acquire toxoplasmosis in small ruminants [18, 31, 47].

Sheep and goats situated at an altitude higher than 100 m above sea level have a higher risk of *T. gondii* infection than those at lower altitudes [49]. The elevation of the Yabello district ranges from 350 to 1857 masl. On average, the altitude of the study area ranges from 850 to 1650 masl in lowlands and midlands, respectively. Due to the sloppy nature of midland areas, viable *T. gondii* oocysts may be carried down by flooding or wind to the plain lowlands surrounding the central midland of the Yabello district. Parallel to this, the abundance of intermediate hosts (farm animals) in lowland areas more than in central midland of the district may be facilitated readily ingestion of viable *T. gondii* oocysts arrived from a higher altitude before desiccation commenced. Soil depletion at a higher altitude by flooding and subsequent deposition at the bottom valley may enhance the moisture content of the soil. Moreover, the higher density of cats in the rural PAs of lowland than periurban PAs of midland in the study district may be attributed to the result. Climate variation is not a predictor for variation in seroprevalence of toxoplasmosis but the difference in management and environmental hygiene in each location is a predictor [28].

In this study, water was found as a vehicle for the transmission of *T. gondii* in animals. In Borana pastoral area, animals were totally kept under an extensive management system, and the available water source was only either lakes or wells. The odds of acquiring *T. gondii* infection were higher in sheep drinking lake water (AOR = 1.93; CI: 1.011–3.698; *P* = 0.046.). In goats, also higher odds of *T. gondii* infection were also encountered from those who managed to drink lake water (AOR = 2.96; CI: 1.297–6.771; *P* = 0.010). This result was in agreement with Ajmal who reported the higher prevalence of *T. gondii* oocyst from lake water than water reservoirs [50]. Water has been suggested as a source of *T. gondii* infection in different countries such as Brazil, Turkey, São Tomé, and Príncipe [51, 52]. The oocysts of *T. gondii* have been isolated from drinking water in Yemen [53]. Small ruminants are mostly infected by *T. gondii* via contaminated feed and water [47].

Variations in seropositivity of *T. gondii* between animals managed to drink lake and wells water in the current study could be due to the difference in the accessibility of water by both wild and domestic felids. Water contamination would be higher in lakes than wells since lakes are fed and drained by rivers and streams which may carry *T. gondii* oocysts and dead bodies from the surface. Wells water is underground spring water accessed by digging a very narrow and deep hole that cannot be drained by running water and reached by running water. In Borana pastoral community, wells water is exclusively reserved for human consumption and improved more than lakes. However, during drying up of lakes and rivers, it is used for both animals and humans by manually drawing water from a deep hole using a half-cut plastic water container. An unimproved water source induces a twofold risk of *T. gondii* compared to an improved water source [54]. The depth of wells and lakes may also decrease access to oocysts as they may found more likely at the bottom of water due to their specific gravity [50].

Regarding the flock size, there was no significant association with seropositivity of *T. gondii* in sheep (*P* = 0.274). This finding was in contrast to outcome from Southern Ethiopia which found higher odds of *T. gondii* infection in sheep from the small flock which was justified as localized grazing range to home and farms where domestic cats are abundant whereas large flocks graze over a wide area unreached by domestic cats [40]. Similarly, in goats, there was no significant association between flock size and *T. gondii* infection (*P* = 0.132). This finding is inconsistent with other results that argued that the large flocks are usually managed under an extensive management system and their chance of getting toxoplasmosis is higher than intensively managed small flocks [55, 56]. In Borana, pastoral area mixing of the flocks is common because of manpower limitation to handle a large number of livestock owned by individuals. As a result, animals in a particular neighbor is usually merged and kept together which may equalize the occurrence of flock-related events.

Concerning cat contact and seropositivity of animals, it was expected that animals owned by pastoralists having domestic cats had higher odds of *T. gondii i*nfection. However, reversely, there was no significant association between *T. gondii* infection and the presence of cats in sheep (*P* = 0.350) and goats (*P* = 0.105). This result was conceded with the finding that argued that the presence of domestic cats might not an independent predictor of *T. gondii* infection since stray and wild cats may contaminate grazing land [57]. In fact, the presence of cats is crucial in the life cycle of *T. gondii* and is significantly associated with *T. gondii* infection [47]. However, in addition to the presence, cats must be carried with *T. gondii.* Moreover, in the current area, animals are entirely kept under an extensive management system (outdoor) and may acquire *T. gondii* oocysts from pasture or water contaminated by feral cats and wild felids. In Italy, the access of stray cats to animals' water and the presence of wild felids were potential risk factors for the seroprevalence of *T. gondii* in dairy sheep flocks [58]. The presence of free-roaming cats is an important risk factor for the transmission of the infection in goats [59].

*T. gondii* is an important cause of abortion and neonatal mortality in sheep and goats [47]. In the present study, the correlation between the history of abortion in flocks and seropositivity to *T. gondii* antibody was assessed. However, this association was found not significant in sheep (*P* = 0.143). Reversely, in goats, there was a significant association between *T. gondii* seropositivity and the history of abortion in the flock (AOR = 2.42; CI: 1.242–4.711; *P* = 0.009). There is a significant association between *T. gondii* seropositivity and the history of abortion in the flock of sheep and goats [60]. However, an insignificant association between *T. gondii* seropositivity and the history of abortion in sheep and goats was also reported [57]. The severity of abortion induced by *T. gondii* infection depends on the time of infection (severe in early gestation) and immune status of ewes and does [61]. On the other hand, abortion in small ruminants is multifactorial; there are more reasons for abortion other than toxoplasmosis [48].

Regarding the awareness level of the community and risk factors of toxoplasmosis, 90% of the respondents were uneducated and rural residents. Knowledge about toxoplasmosis and cat risk to animal and human health was very poor. But felids play a key role in the transmission of *T. gondii* [62]. In this area, dogs are inseparable from the flock throughout their life and went to pasture and water together with the flock, guard against predators, and consequently contaminate water and pasture [22]. On the other hand, hygienic water sources are scarce; only 5% of respondents had access to pipe water. The oocysts of *T. gondii* have been isolated from drinking water and indicated that unimproved water sources had a twofold higher risk of infected *T. gondii* infection compared with improved water sources [53, 54].

Regarding consumption habits, 75% consume raw milk of sheep and goats which may increase the risk of acquiring toxoplasmosis in pastoralists. *T. gondii* tachyzoites have been detected in the milk of ewes and goats [63]. Human cases of toxoplasmosis have been linked directly to the consumption of raw goat milk [64]. Mainly unpasteurized milk from acutely diseased goats is an important source of *T. gondii* infection in children [30]. Concerning fetus handling trend, 63% of flock owners had encountered at least one case of abortion and 67% of respondents handled aborted fetuses in an unhygienic manner. *T. gondii* is an important cause of abortion and neonatal mortality in small ruminants, and burying and burning of aborted fetuses were suggested as methods of control of disease transmission [65]. The worst, in pastoral areas, routine husbandry activities like sick animal care, milking, and cleaning barns are exclusively undertaken by women while males control over activities performed outdoor: looking after livestock, pasture, and water management. This scenario may exacerbate the risk of *T. gondii* infection in pregnant women of the pastoral community.

## 5. Conclusions

This is the first serological survey of toxoplasmosis in small ruminants of the Borana pastoral area of Southern Ethiopia. The results of the present study indicated that toxoplasmosis is highly prevalent in small ruminants. Collectively, age, sex, water source, and altitude are significant predictors of *T. gondii* seropositivity in small ruminants. Almost all of the interviewed pastoralists had no knowledge about toxoplasmosis and extremely exposed to a cluster of risk factors that could induce toxoplasmosis. Based on the above conclusion, the following recommendations are forwarded:

- Healthy education about toxoplasmosis and exposing risk factors such as consumption of raw milk and meat, improper fetus, and dead body handling should be provided for the community in this area
- Provision of hygienic water for human and animals and sanitary measures of water sources are also of paramount importance to tackle the economic and public health consequences of *T. gondii* infection
- Furthermore, further studies using confirmatory diagnosis tests that encompass both animals and humans should be conducted to obtain a real figure of toxoplasmosis distribution in pastoral areas

## Figures and Tables

**Table 1:** Overall seroprevalence of *T. gondii* infection in small ruminants in six PAs of Yabello.

PA	No. tested	No. positive	Seroprevalence (%)
Dida Yabello	70	31	44.3
Haro Bake	64	51	79.7
Darito	68	49	72
Areri	65	25	38.5
Obdaya	67	11	16.4
Cholkasa	66	44	66.7
Total	400	211	52.8

PA = pastoral association.

**Table 2:** Seroprevalence of *T. gondii* infection in sheep and goats of studied PAs of the Yabello district.

PA	Sheep		Goat	
	No. examined	No. positive (seroprevalence)	No. examined	No. positive (seroprevalence)
Dida Yabello	29	18 (62%)	41	13 (31.7%)
Haro Bake	24	18 (75%)	40	33 (82.5%)
Darito	29	25 (86.2%)	39	24 (61.5%)
Areri	45	21 (46.7%)	20	4 (20%)
Obdaya	32	3 (9.4%)	35	8 (22.8%)
Cholkasa	38	29 (76.3%)	28	15 (53.6%)
Total	197	114 (57.8%)	203	97 (47.8%)

PA = pastoral association.

**Table 3:** Seroprevalence of *T. gondii* antibody and logistic regression output of risk factors and variables for sheep.

Risk factor/variable					Univariable		Multivariable	
		NE	NP	SP%	COR (95% CI)	*P* value	AOR (95% CI)	*P* value
Species	Sheep	197	114	57.8	1.50 (1.011–2.227)	0.044		
	Goat	203	97	47.8	1			
	Total	400	211	52.8				
Sex	Female	119	80	67.2	2.65 (1.473–4.784)	0.001	2.45 (1.313–4.568)	0.005
	Male	78	34	43.6	1		1	
Age	Adult	158	98	62	2.35 (1.149–4.797)	0.019	2.13 (1.137–5.618)	0.023
	Young	39	16	41				
Ecology	Midland	89	38	42.7	1	<0.001	1	0.013
	Lowland	108	76	70.4	3.19 (1.769–5.745)		2.28 (1.190–4.356)	
Water	Well	92	41	44.5	1		1	0.046
	Lake	105	73	69.5	2.84 (1.582–5.091)	<0.001	1.93 (1.011–3.698)	
Cat contact	Yes	133	80	60.2	1.33 (0.730–2.430)	0.350		
	No	64	34	53	1			
Flock size	Large	111	68	61.3	1.37 (0.777–2.433)	0.274		
	Small	86	46	53.5	1			
Abortion history	Yes	137	74	54	1.70 (0.904–3.207)	0.100	1.69 (0.837–3.431)	0.143
	No	60	40	66.7	1		1	

NE = number examined, NP = number positive, SP = seroprevalence, COR = crude odds ratio, AOR = adjusted odds ratio, CI = confidence interval.

**Table 4:** Seroprevalence of *T. gondii* antibody and logistic regression analysis output of risk factors and variables for goats.

Risk factor/variable					Univariable		Multivariable	
		NE	NP	SP%	COR (95% CI)	*P* value	AOR (95% CI)	*P* value
Sex	Female	154	68	44.2	1	0.069		
	Male	49	29	59.2	1.83 (0.955–3.521)			

Age	Adult	151	82	54.3	2.93 (1.485–5.786)	0.002	2.15 (1.009–4.579)	0.047
	Young	52	15	28.8	1			

Ecology	Midland	80	17	21.3	1		1	0.007
	Lowland	123	80	65	6.89 (3.594–13.225)	<0.001	3.27 (1.386–7.723)	

Water	Well	97	26	26.8	1		1	0. 010
	Lake	106	71	77	5.54 (3.026–10.142)	<0.001	2.96 (1.297–6.771)	

Cat contact	Yes	143	79	55.2	2.88 (1.514–5.479)	0.001	1.86 (0.878–3.933)	0.105
	No	60	18	30	1		1	

Flock size	Large	134	68	50.7	1.421 (0.791–2.553)	0.240	1.74 (0.845–3.601)	0.132
	Small	69	29	42	1			

Abortion history	Yes	114	64	56.	2.17 (1.232–3.831)	0.007	2.42 (1.242–4.711)	0.009
	No	89	33	37	1		1	

NE = number examined, NP = number positive, SP = seroprevalence, COR = crude odds ratio, AOR = adjusted odds ratio, CI = confidence interval.

**Table 5:** Summary of the findings of the questionnaire survey regarding possible risk factors and awareness level of the community about toxoplasmosis in the Yabello district.

Variables	Category	Frequency (%) (*n* = 100)	Toxoplasmosis knowledge	
			Yes	No
Place	Urban	10 (10%)	3 (30%)	7 (70%)
	Rural	90 (90%)	0 (0%)	90 (100%)
	Total	100 (100%)	3 (3%)	97 (97%)

Sex	Male	67 (67%)	3 (4.5%)	65 (95.5%)
	Female	23 (23%)	0 (0%)	23 (100%)

Age	15–24	25 (25%)	0 (0%)	25 (100%)
	25–36	43 (43%)	3 (7%)	40 (93%)
	37–48	19 (19%)	0 (0%)	19 (100%)
	>48	13 (13%)	0 (0%)	13 (100%)

Religion	Wakefata	75 (75%)	1 (3.3%)	74 (96.7%)
	Muslim	15 (15%)	1 (6.7%)	14 (93.3%)
	Protestant	10 (10%)	1 (10%)	9 (90%)

Education	Uneducated	86 (86%)	0 (0%)	86 (100%)
	Primary school	6 (6%)	0 (0%)	6 (100%)
	Secondary school	2 (2%)	0 (0%)	2 (100%)
	Tertiary school	6 (6%)	3 (50%)	3 (50%)

Water source	Pipe water	5 (5%)	3 (60%)	2 (40%)
	Well	23 (23%)	0 (0%)	23 (100%)
	Lake	33 (33%)	0 (0%)	33 (100%)
	Mixed	39 (39%)	0 (0%)	39 (100%)

Raw milk consumption	Yes	75 (75%)	0 (0%)	75 (100%)
	No	25 (25%)	3 (12%)	22 (88%)

Raw meat consumption	Yes	80 (80%)	0 (0%)	80 (100%)
	No	20 (20%)	3 (15%)	3 (15%)

Fetus handling	Hand wash	33 (33%)	3 (9%)	30 (91%)
	No hand wash	3 (3%)	0 (0%)	3 (100%)
	Dry hand by cloth	63 (63%)	0 (0%)	63 (100%)

Presence of domestic cat	Yes	70 (70%)	2 (2.9%)	68 (97.1%)
	No	30 (30%)	1 (3.3%)	29 (96.7%)

Cat risk to human health	Yes	53 (53%)	3 (5.5%)	50 (94.5%)
	No	47 (47%)	0 (0%)	47 (100%)

Cat risk to animal health	Yes	20 (20%)	3 (15%)	17 (75%)
	No	80 (80%)	0 (0%)	80 (100%)

Abortion in flock	Yes	63 (63%)	1 (1.6%)	62 (98.4%)
	No	27 (27%)	2 (7.4%)	25 (92.6%)

## Data Availability

The data are accessible on request from the corresponding author.
